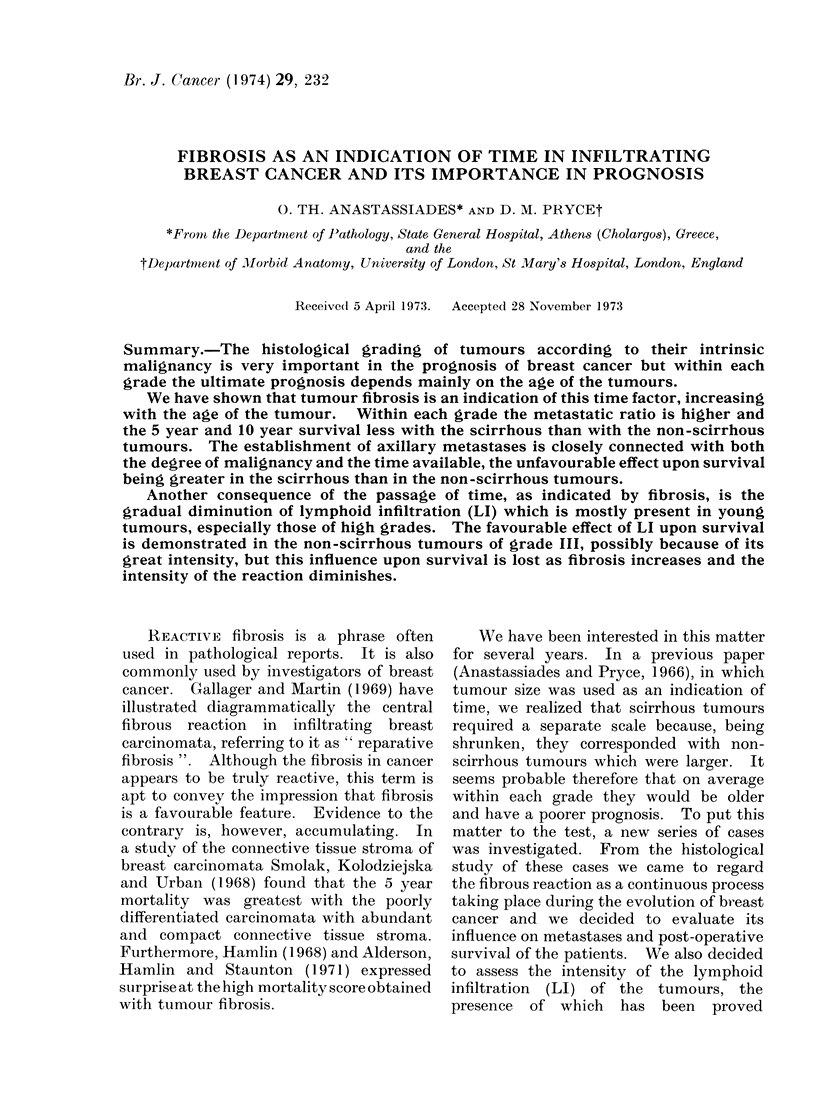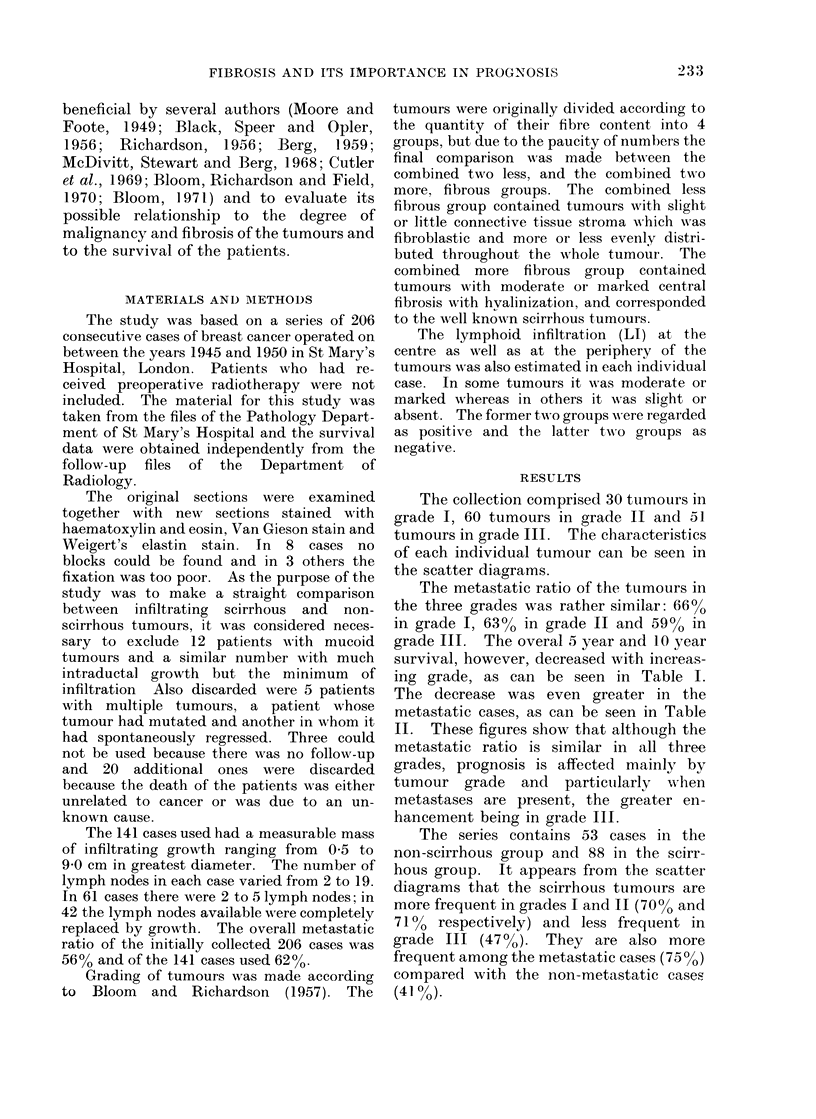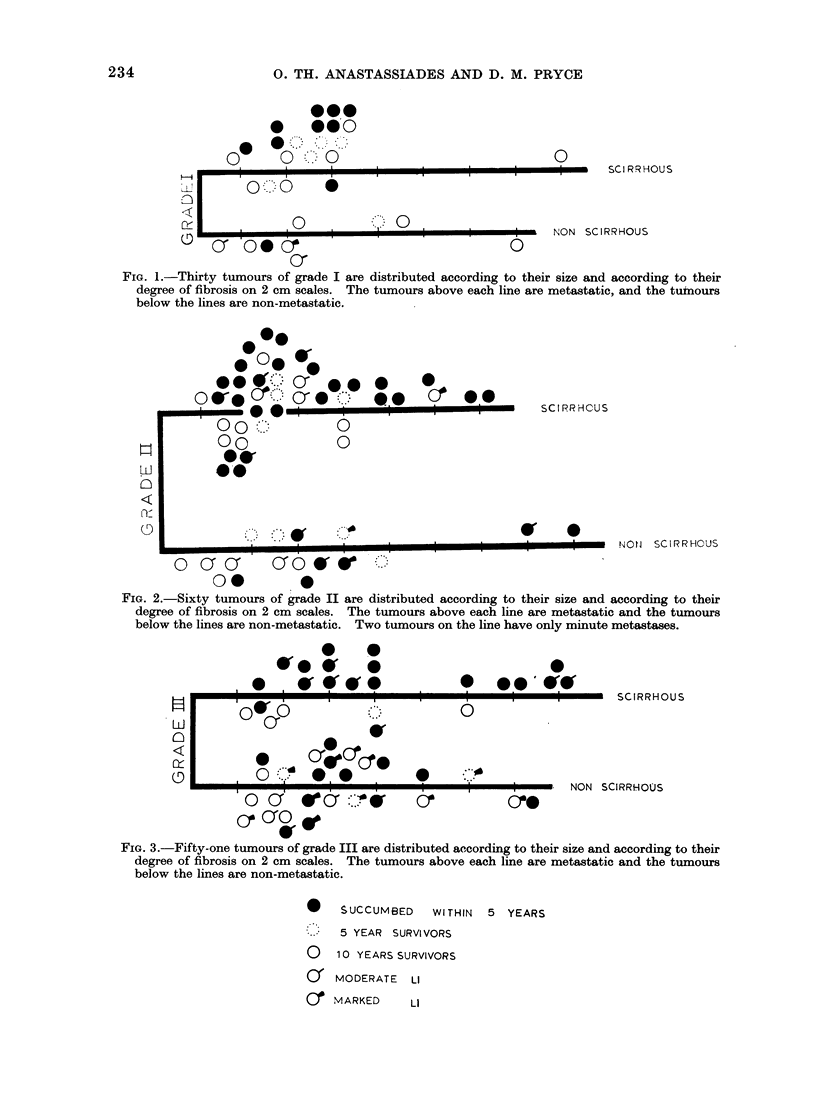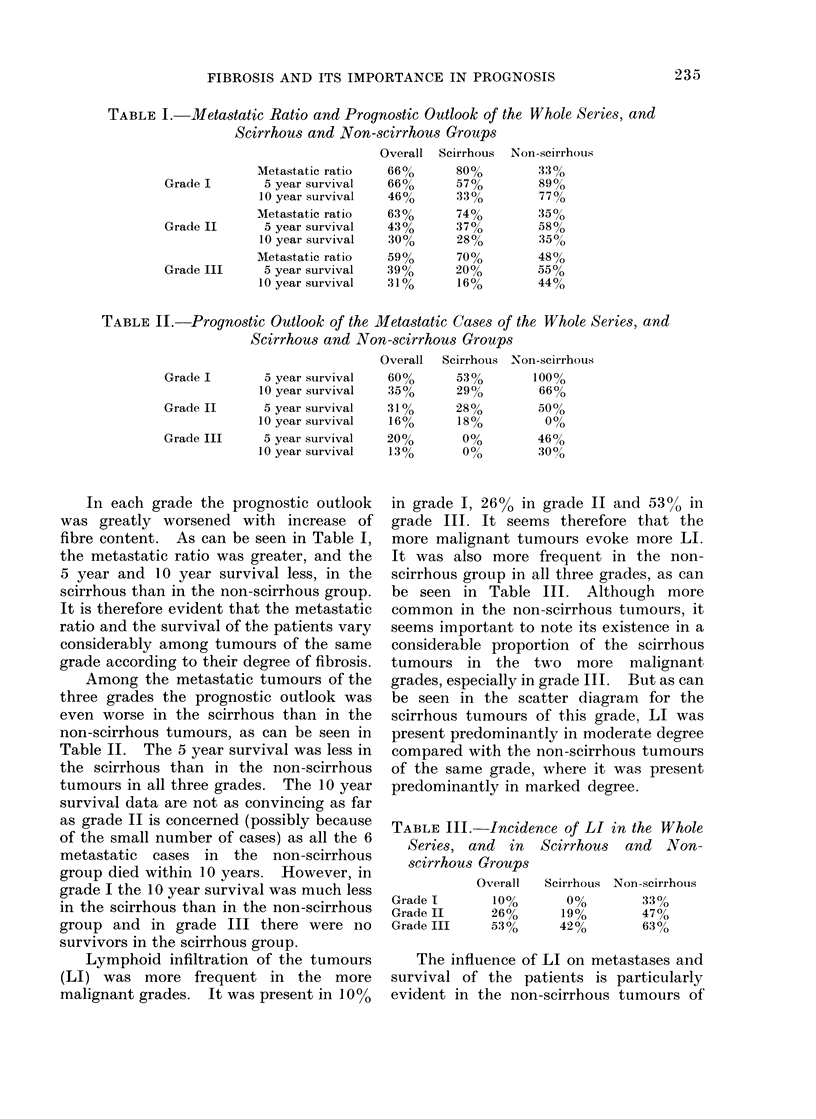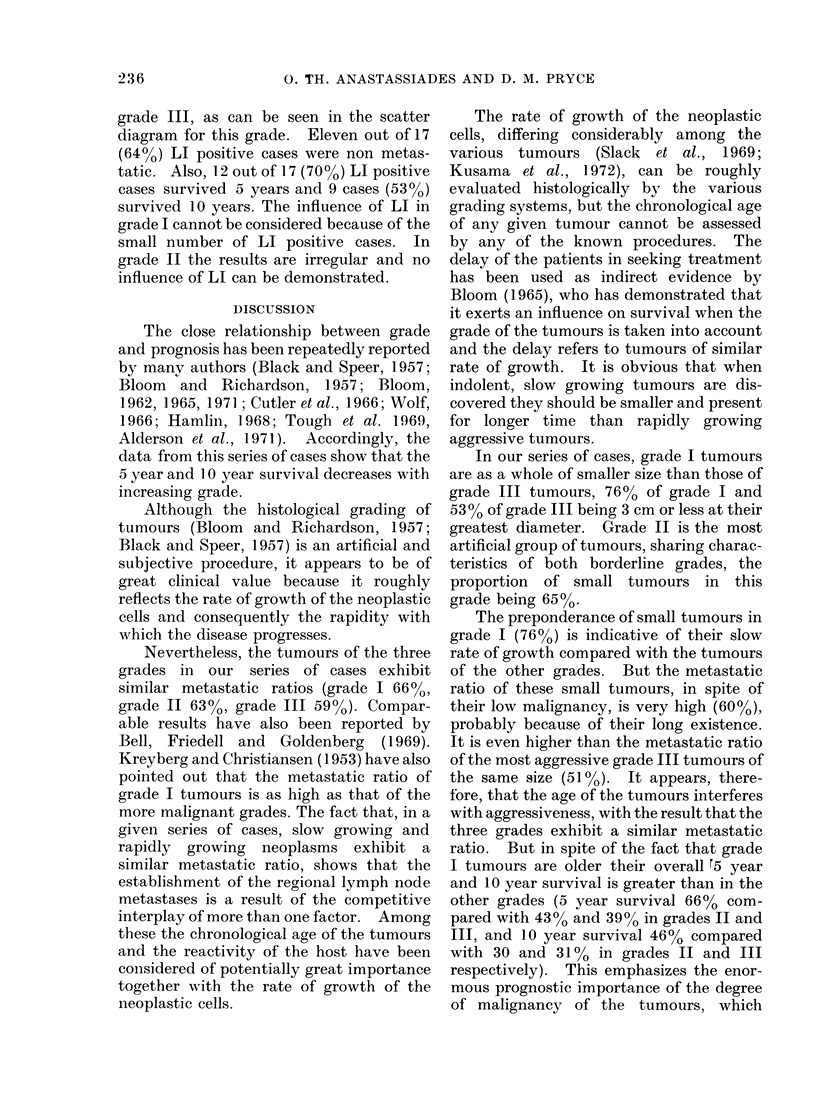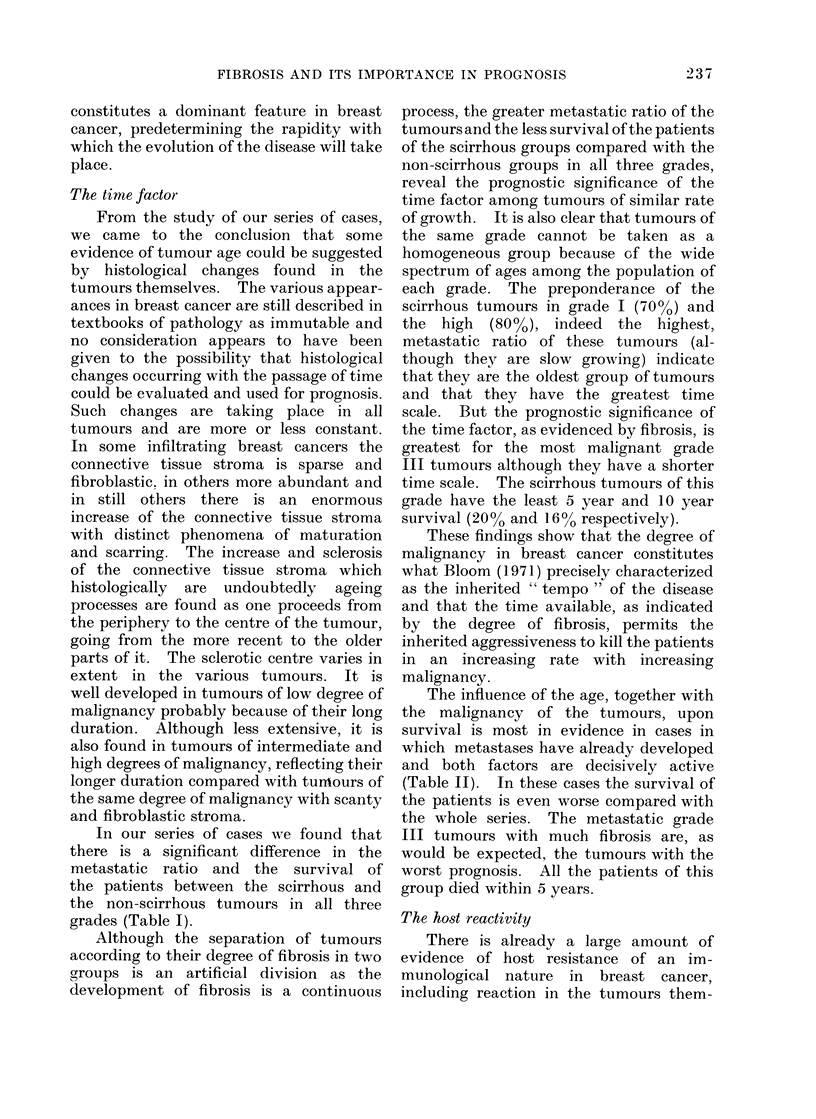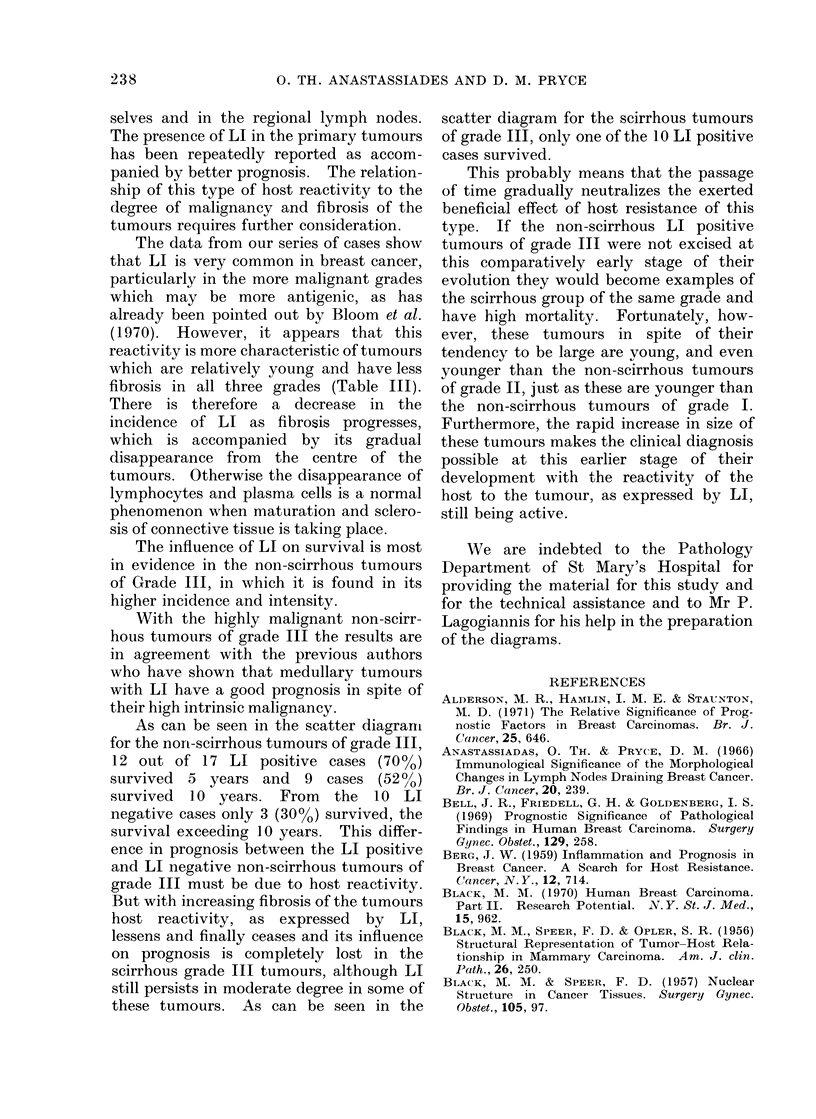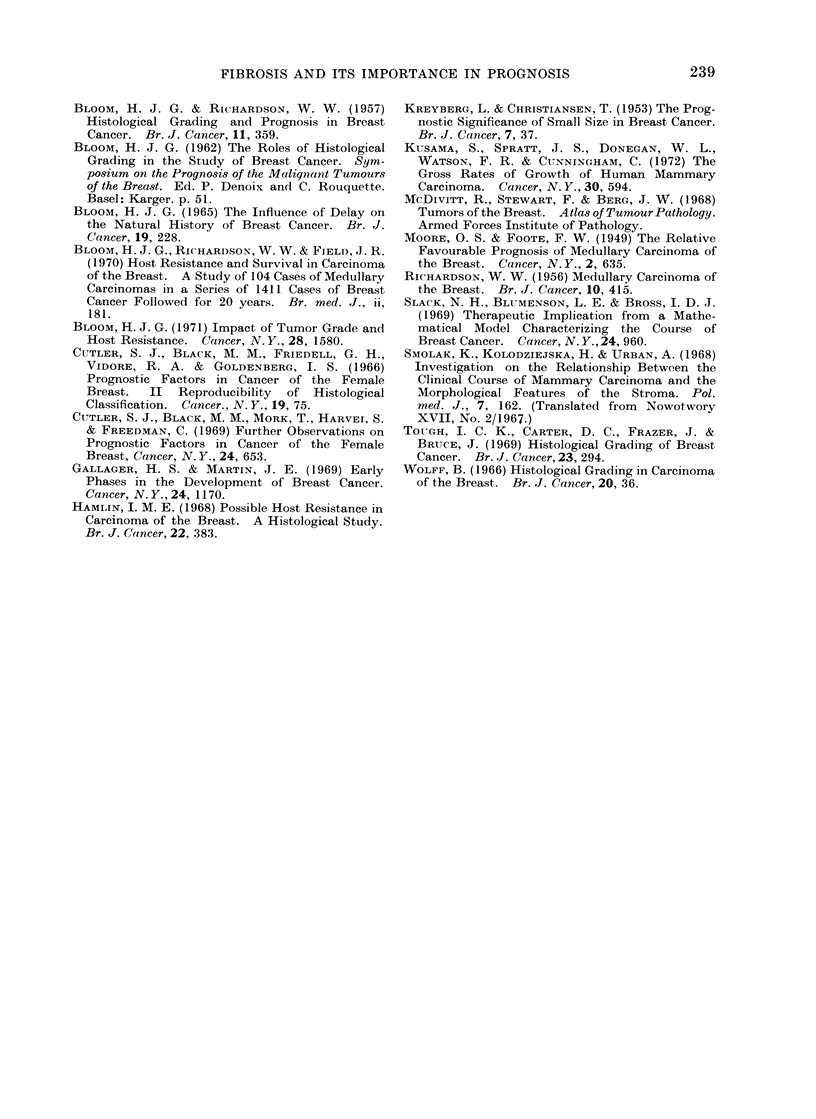# Fibrosis as an Indication of Time in Infiltrating Breast Cancer and its Importance in Prognosis

**DOI:** 10.1038/bjc.1974.62

**Published:** 1974-03

**Authors:** O. Th. Anastassiades, D. M. Pryce

## Abstract

The histological grading of tumours according to their intrinsic malignancy is very important in the prognosis of breast cancer but within each grade the ultimate prognosis depends mainly on the age of the tumours.

We have shown that tumour fibrosis is an indication of this time factor, increasing with the age of the tumour. Within each grade the metastatic ratio is higher and the 5 year and 10 year survival less with the scirrhous than with the non-scirrhous tumours. The establishment of axillary metastases is closely connected with both the degree of malignancy and the time available, the unfavourable effect upon survival being greater in the scirrhous than in the non-scirrhous tumours.

Another consequence of the passage of time, as indicated by fibrosis, is the gradual diminution of lymphoid infiltration (LI) which is mostly present in young tumours, especially those of high grades. The favourable effect of LI upon survival is demonstrated in the non-scirrhous tumours of grade III, possibly because of its great intensity, but this influence upon survival is lost as fibrosis increases and the intensity of the reaction diminishes.


*Br. J. Cancer* (1974) **29**, 232

### FIBROSIS AS AN INDICATION OF TIME IN INFILTRATING BREAST CANCER AND ITS IMPORTANCE IN PROGNOSIS

O. TH. ANASTASSIADES* AND D. M. PRYCE†

**From the Department of Pathology, State General Hospital, Athens (Cholargos), Greece,*
*and the*
†*Department of Morbid Anatomy, University of London, St Mary's Hospital, London, England*

Received 5 April 1973. Accepted 28 November 1973

**Summary.—The histological grading of tumours according to their intrinsic malignancy is very important in the prognosis of breast cancer but within each grade the ultimate prognosis depends mainly on the age of the tumours.**

**We have shown that tumour fibrosis is an indication of this time factor, increasing with the age of the tumour. Within each grade the metastatic ratio is higher and the 5 year and 10 year survival less with the scirrhous than with the non-scirrhous tumours. The establishment of axillary metastases is closely connected with both the degree of malignancy and the time available, the unfavourable effect upon survival being greater in the scirrhous than in the non-scirrhous tumours.**

**Another consequence of the passage of time, as indicated by fibrosis, is the gradual diminution of lymphoid infiltration (LI) which is mostly present in young tumours, especially those of high grades. The favourable effect of LI upon survival is demonstrated in the non-scirrhous tumours of grade III, possibly because of its great intensity, but this influence upon survival is lost as fibrosis increases and the intensity of the reaction diminishes.**

REACTIVE fibrosis is a phrase often used in pathological reports. It is also commonly used by investigators of breast cancer. Gallager and Martin (1969) have illustrated diagrammatically the central fibrous reaction in infiltrating breast carcinomata, referring to it as " reparative fibrosis ". Although the fibrosis in cancer appears to be truly reactive, this term is apt to convey the impression that fibrosis is a favourable feature. Evidence to the contrary is, however, accumulating. In a study of the connective tissue stroma of breast carcinomata Smolak, Kolodziejska and Urban (1968) found that the 5 year mortality was greatest with the poorly differentiated carcinomata with abundant and compact connective tissue stroma. Furthermore, Hamlin (1968) and Alderson, Hamlin and Staunton (1971) expressed surprise at the high mortality score obtained with tumour fibrosis.

We have been interested in this matter for several years. In a previous paper (Anastassiades and Pryce, 1966), in which tumour size was used as an indication of time, we realized that scirrhous tumours required a separate scale because, being shrunken, they corresponded with non-scirrhous tumours which were larger. It seems probable therefore that on average within each grade they would be older and have a poorer prognosis. To put this matter to the test, a new series of cases was investigated. From the histological study of these cases we came to regard the fibrous reaction as a continuous process taking place during the evolution of breast cancer and we decided to evaluate its influence on metastases and post-operative survival of the patients. We also decided to assess the intensity of the lymphoid infiltration (LI) of the tumours, the presence of which has been proved beneficial by several authors (Moore and Foote, 1949; Black, Speer and Opler, 1956; Richardson, 1956; Berg, 1959; McDivitt, Stewart and Berg, 1968; Cutler *et al.*, 1969; Bloom, Richardson and Field, 1970; Bloom, 1971) and to evaluate its possible relationship to the degree of malignancy and fibrosis of the tumours and to the survival of the patients.

### MATERIALS AND METHODS

The study was based on a series of 206 consecutive cases of breast cancer operated on between the years 1945 and 1950 in St Mary's Hospital, London. Patients who had received preoperative radiotherapy were not included. The material for this study was taken from the files of the Pathology Department of St Mary's Hospital and the survival data were obtained independently from the follow-up files of the Department of Radiology.

The original sections were examined together with new sections stained with haematoxylin and eosin, Van Gieson stain and Weigert's elastin stain. In 8 cases no blocks could be found and in 3 others the fixation was too poor. As the purpose of the study was to make a straight comparison between infiltrating scirrhous and non-scirrhous tumours, it was considered necessary to exclude 12 patients with mucoid tumours and a similar number with much intraductal growth but the minimum of infiltration Also discarded were 5 patients with multiple tumours, a patient whose tumour had mutated and another in whom it had spontaneously regressed. Three could not be used because there was no follow-up and 20 additional ones were discarded because the death of the patients was either unrelated to cancer or was due to an unknown cause.

The 141 cases used had a measurable mass of infiltrating growth ranging from 0·5 to 9·0 cm in greatest diameter. The number of lymph nodes in each case varied from 2 to 19. In 61 cases there were 2 to 5 lymph nodes; in 42 the lymph nodes available were completely replaced by growth. The overall metastatic ratio of the initially collected 206 cases was 56% and of the 141 cases used 62%.

Grading of tumours was made according to Bloom and Richardson (1957). The tumours were originally divided according to the quantity of their fibre content into 4 groups, but due to the paucity of numbers the final comparison was made between the combined two less, and the combined two more, fibrous groups. The combined less fibrous group contained tumours with slight or little connective tissue stroma which was fibroblastic and more or less evenly distributed throughout the whole tumour. The combined more fibrous group contained tumours with moderate or marked central fibrosis with hyalinization, and corresponded to the well known scirrhous tumours.

The lymphoid infiltration (LI) at the centre as well as at the periphery of the tumours was also estimated in each individual case. In some tumours it was moderate or marked whereas in others it was slight or absent. The former two groups were regarded as positive and the latter two groups as negative.

### RESULTS

The collection comprised 30 tumours in grade I, 60 tumours in grade II and 51 tumours in grade III. The characteristics of each individual tumour can be seen in the scatter diagrams.

The metastatic ratio of the tumours in the three grades was rather similar: 66% in grade I, 63% in grade II and 59% in grade III. The overal 5 year and 10 year survival, however, decreased with increasing grade, as can be seen in Table I. The decrease was even greater in the metastatic cases, as can be seen in Table II. These figures show that although the metastatic ratio is similar in all three grades, prognosis is affected mainly by tumour grade and particularly when metastases are present, the greater enhancement being in grade III.

The series contains 53 cases in the non-scirrhous group and 88 in the scirrhous group. It appears from the scatter diagrams that the scirrhous tumours are more frequent in grades I and II (70% and 71% respectively) and less frequent in grade III (47%). They are also more frequent among the metastatic cases (75%) compared with the non-metastatic cases (41%).

FIG. 1.—Thirty tumours of grade I are distributed according to their size and according to their degree of fibrosis on 2 cm scales. The tumours above each line are metastatic, and the tumours below the lines are non-metastatic.

FIG. 2.—Sixty tumours of grade II are distributed according to their size and according to their degree of fibrosis on 2 cm scales. The tumours above each line are metastatic and the tumours below the lines are non-metastatic. Two tumours on the line have only minute metastases.

FIG. 3.—Fifty-one tumours of grade III are distributed according to their size and according to their degree of fibrosis on 2 cm scales. The tumours above each line are metastatic and the tumours below the lines are non-metastatic.

TABLE I.—*Metastatic Ratio and Prognostic Outlook of the Whole Series, and Scirrhous and Non-scirrhous Groups*

| | | Overall | Scirrhous | Non-scirrhous |
|---|---|---|---|---|
| | Metastatic ratio | 66% | 80% | 33% |
| Grade I | 5 year survival | 66% | 57% | 89% |
| | 10 year survival | 46% | 33% | 77% |
| | Metastatic ratio | 63% | 74% | 35% |
| Grade II | 5 year survival | 43% | 37% | 58% |
| | 10 year survival | 30% | 28% | 35% |
| | Metastatic ratio | 59% | 70% | 48% |
| Grade III | 5 year survival | 39% | 20% | 55% |
| | 10 year survival | 31% | 16% | 44% |

TABLE II.—*Prognostic Outlook of the Metastatic Cases of the Whole Series, and Scirrhous and Non-scirrhous Groups*

| | | Overall | Scirrhous | Non-scirrhous |
|---|---|---|---|---|
| Grade I | 5 year survival | 60% | 53% | 100% |
| | 10 year survival | 35% | 29% | 66% |
| Grade II | 5 year survival | 31% | 28% | 50% |
| | 10 year survival | 16% | 18% | 0% |
| Grade III | 5 year survival | 20% | 0% | 46% |
| | 10 year survival | 13% | 0% | 30% |

In each grade the prognostic outlook was greatly worsened with increase of fibre content. As can be seen in Table I, the metastatic ratio was greater, and the 5 year and 10 year survival less, in the scirrhous than in the non-scirrhous group. It is therefore evident that the metastatic ratio and the survival of the patients vary considerably among tumours of the same grade according to their degree of fibrosis.

Among the metastatic tumours of the three grades the prognostic outlook was even worse in the scirrhous than in the non-scirrhous tumours, as can be seen in Table II. The 5 year survival was less in the scirrhous than in the non-scirrhous tumours in all three grades. The 10 year survival data are not as convincing as far as grade II is concerned (possibly because of the small number of cases) as all the 6 metastatic cases in the non-scirrhous group died within 10 years. However, in grade I the 10 year survival was much less in the scirrhous than in the non-scirrhous group and in grade III there were no survivors in the scirrhous group.

Lymphoid infiltration of the tumours (LI) was more frequent in the more malignant grades. It was present in 10% in grade I, 26% in grade II and 53% in grade III. It seems therefore that the more malignant tumours evoke more LI. It was also more frequent in the non-scirrhous group in all three grades, as can be seen in Table III. Although more common in the non-scirrhous tumours, it seems important to note its existence in a considerable proportion of the scirrhous tumours in the two more malignant grades, especially in grade III. But as can be seen in the scatter diagram for the scirrhous tumours of this grade, LI was present predominantly in moderate degree compared with the non-scirrhous tumours of the same grade, where it was present predominantly in marked degree.

TABLE III.—*Incidence of LI in the Whole Series, and in Scirrhous and Non-scirrhous Groups*

| | Overall | Scirrhous | Non-scirrhous |
|---|---|---|---|
| Grade I | 10% | 0% | 33% |
| Grade II | 26% | 19% | 47% |
| Grade III | 53% | 42% | 63% |

The influence of LI on metastases and survival of the patients is particularly evident in the non-scirrhous tumours of grade III, as can be seen in the scatter diagram for this grade. Eleven out of 17 (64%) LI positive cases were non metastatic. Also, 12 out of 17 (70%) LI positive cases survived 5 years and 9 cases (53%) survived 10 years. The influence of LI in grade I cannot be considered because of the small number of LI positive cases. In grade II the results are irregular and no influence of LI can be demonstrated.

### DISCUSSION

The close relationship between grade and prognosis has been repeatedly reported by many authors (Black and Speer, 1957; Bloom and Richardson, 1957; Bloom, 1962, 1965, 1971; Cutler *et al.*, 1966; Wolf, 1966; Hamlin, 1968; Tough *et al.* 1969, Alderson *et al.*, 1971). Accordingly, the data from this series of cases show that the 5 year and 10 year survival decreases with increasing grade.

Although the histological grading of tumours (Bloom and Richardson, 1957; Black and Speer, 1957) is an artificial and subjective procedure, it appears to be of great clinical value because it roughly reflects the rate of growth of the neoplastic cells and consequently the rapidity with which the disease progresses.

Nevertheless, the tumours of the three grades in our series of cases exhibit similar metastatic ratios (grade I 66%, grade II 63%, grade III 59%). Comparable results have also been reported by Bell, Friedell and Goldenberg (1969). Kreyberg and Christiansen (1953) have also pointed out that the metastatic ratio of grade I tumours is as high as that of the more malignant grades. The fact that, in a given series of cases, slow growing and rapidly growing neoplasms exhibit a similar metastatic ratio, shows that the establishment of the regional lymph node metastases is a result of the competitive interplay of more than one factor. Among these the chronological age of the tumours and the reactivity of the host have been considered of potentially great importance together with the rate of growth of the neoplastic cells.

The rate of growth of the neoplastic cells, differing considerably among the various tumours (Slack *et al.*, 1969; Kusama *et al.*, 1972), can be roughly evaluated histologically by the various grading systems, but the chronological age of any given tumour cannot be assessed by any of the known procedures. The delay of the patients in seeking treatment has been used as indirect evidence by Bloom (1965), who has demonstrated that it exerts an influence on survival when the grade of the tumours is taken into account and the delay refers to tumours of similar rate of growth. It is obvious that when indolent, slow growing tumours are discovered they should be smaller and present for longer time than rapidly growing aggressive tumours.

In our series of cases, grade I tumours are as a whole of smaller size than those of grade III tumours, 76% of grade I and 53% of grade III being 3 cm or less at their greatest diameter. Grade II is the most artificial group of tumours, sharing characteristics of both borderline grades, the proportion of small tumours in this grade being 65%.

The preponderance of small tumours in grade I (76%) is indicative of their slow rate of growth compared with the tumours of the other grades. But the metastatic ratio of these small tumours, in spite of their low malignancy, is very high (60%), probably because of their long existence. It is even higher than the metastatic ratio of the most aggressive grade III tumours of the same size (51%). It appears, therefore, that the age of the tumours interferes with aggressiveness, with the result that the three grades exhibit a similar metastatic ratio. But in spite of the fact that grade I tumours are older their overall 5 year and 10 year survival is greater than in the other grades (5 year survival 66% compared with 43% and 39% in grades II and III, and 10 year survival 46% compared with 30 and 31% in grades II and III respectively). This emphasizes the enormous prognostic importance of the degree of malignancy of the tumours, which constitutes a dominant feature in breast cancer, predetermining the rapidity with which the evolution of the disease will take place.

*The time factor*

From the study of our series of cases, we came to the conclusion that some evidence of tumour age could be suggested by histological changes found in the tumours themselves. The various appearances in breast cancer are still described in textbooks of pathology as immutable and no consideration appears to have been given to the possibility that histological changes occurring with the passage of time could be evaluated and used for prognosis. Such changes are taking place in all tumours and are more or less constant. In some infiltrating breast cancers the connective tissue stroma is sparse and fibroblastic, in others more abundant and in still others there is an enormous increase of the connective tissue stroma with distinct phenomena of maturation and scarring. The increase and sclerosis of the connective tissue stroma which histologically are undoubtedly ageing processes are found as one proceeds from the periphery to the centre of the tumour, going from the more recent to the older parts of it. The sclerotic centre varies in extent in the various tumours. It is well developed in tumours of low degree of malignancy probably because of their long duration. Although less extensive, it is also found in tumours of intermediate and high degrees of malignancy, reflecting their longer duration compared with tumours of the same degree of malignancy with scanty and fibroblastic stroma.

In our series of cases we found that there is a significant difference in the metastatic ratio and the survival of the patients between the scirrhous and the non-scirrhous tumours in all three grades (Table I).

Although the separation of tumours according to their degree of fibrosis in two groups is an artificial division as the development of fibrosis is a continuous process, the greater metastatic ratio of the tumours and the less survival of the patients of the scirrhous groups compared with the non-scirrhous groups in all three grades, reveal the prognostic significance of the time factor among tumours of similar rate of growth. It is also clear that tumours of the same grade cannot be taken as a homogeneous group because of the wide spectrum of ages among the population of each grade. The preponderance of the scirrhous tumours in grade I (70%) and the high (80%), indeed the highest, metastatic ratio of these tumours (although they are slow growing) indicate that they are the oldest group of tumours and that they have the greatest time scale. But the prognostic significance of the time factor, as evidenced by fibrosis, is greatest for the most malignant grade III tumours although they have a shorter time scale. The scirrhous tumours of this grade have the least 5 year and 10 year survival (20% and 16% respectively).

These findings show that the degree of malignancy in breast cancer constitutes what Bloom (1971) precisely characterized as the inherited " tempo " of the disease and that the time available, as indicated by the degree of fibrosis, permits the inherited aggressiveness to kill the patients in an increasing rate with increasing malignancy.

The influence of the age, together with the malignancy of the tumours, upon survival is most in evidence in cases in which metastases have already developed and both factors are decisively active (Table II). In these cases the survival of the patients is even worse compared with the whole series. The metastatic grade III tumours with much fibrosis are, as would be expected, the tumours with the worst prognosis. All the patients of this group died within 5 years.

*The host reactivity*

There is already a large amount of evidence of host resistance of an immunological nature in breast cancer, including reaction in the tumours themselves and in the regional lymph nodes. The presence of LI in the primary tumours has been repeatedly reported as accompanied by better prognosis. The relationship of this type of host reactivity to the degree of malignancy and fibrosis of the tumours requires further consideration.

The data from our series of cases show that LI is very common in breast cancer, particularly in the more malignant grades which may be more antigenic, as has already been pointed out by Bloom *et al.* (1970). However, it appears that this reactivity is more characteristic of tumours which are relatively young and have less fibrosis in all three grades (Table III). There is therefore a decrease in the incidence of LI as fibrosis progresses, which is accompanied by its gradual disappearance from the centre of the tumours. Otherwise the disappearance of lymphocytes and plasma cells is a normal phenomenon when maturation and sclerosis of connective tissue is taking place.

The influence of LI on survival is most in evidence in the non-scirrhous tumours of Grade III, in which it is found in its higher incidence and intensity.

With the highly malignant non-scirrhous tumours of grade III the results are in agreement with the previous authors who have shown that medullary tumours with LI have a good prognosis in spite of their high intrinsic malignancy.

As can be seen in the scatter diagram for the non-scirrhous tumours of grade III, 12 out of 17 LI positive cases (70%) survived 5 years and 9 cases (52%) survived 10 years. From the 10 LI negative cases only 3 (30%) survived, the survival exceeding 10 years. This difference in prognosis between the LI positive and LI negative non-scirrhous tumours of grade III must be due to host reactivity. But with increasing fibrosis of the tumours host reactivity, as expressed by LI, lessens and finally ceases and its influence on prognosis is completely lost in the scirrhous grade III tumours, although LI still persists in moderate degree in some of these tumours. As can be seen in the scatter diagram for the scirrhous tumours of grade III, only one of the 10 LI positive cases survived.

This probably means that the passage of time gradually neutralizes the exerted beneficial effect of host resistance of this type. If the non-scirrhous LI positive tumours of grade III were not excised at this comparatively early stage of their evolution they would become examples of the scirrhous group of the same grade and have high mortality. Fortunately, however, these tumours in spite of their tendency to be large are young, and even younger than the non-scirrhous tumours of grade II, just as these are younger than the non-scirrhous tumours of grade I. Furthermore, the rapid increase in size of these tumours makes the clinical diagnosis possible at this earlier stage of their development with the reactivity of the host to the tumour, as expressed by LI, still being active.

We are indebted to the Pathology Department of St Mary's Hospital for providing the material for this study and for the technical assistance and to Mr P. Lagogiannis for his help in the preparation of the diagrams.

REFERENCES

ALDERSON, M. R., HAMLIN, I. M. E. & STAUNTON, M. D. (1971) The Relative Significance of Prognostic Factors in Breast Carcinomas. *Br. J. Cancer*, **25**, 646.

ANASTASSIADAS, O. TH. & PRYCE, D. M. (1966) Immunological Significance of the Morphological Changes in Lymph Nodes Draining Breast Cancer. *Br. J. Cancer*, **20**, 239.

BELL, J. R., FRIEDELL, G. H. & GOLDENBERG, I. S. (1969) Prognostic Significance of Pathological Findings in Human Breast Carcinoma. *Surgery Gynec. Obstet.*, **129**, 258.

BERG, J. W. (1959) Inflammation and Prognosis in Breast Cancer. A Search for Host Resistance. *Cancer, N.Y.*, **12**, 714.

BLACK, M. M. (1970) Human Breast Carcinoma. Part II. Research Potential. *N.Y. St. J. Med.*, **15**, 962.

BLACK, M. M., SPEER, F. D. & OPLER, S. R. (1956) Structural Representation of Tumor–Host Relationship in Mammary Carcinoma. *Am. J. clin. Path.*, **26**, 250.

BLACK, M. M. & SPEER, F. D. (1957) Nuclear Structure in Cancer Tissues. *Surgery Gynec. Obstet.*, **105**, 97.

BLOOM, H. J. G. & RICHARDSON, W. W. (1957) Histological Grading and Prognosis in Breast Cancer. *Br. J. Cancer*, **11**, 359.

BLOOM, H. J. G. (1962) The Roles of Histological Grading in the Study of Breast Cancer. *Symposium on the Prognosis of the Malignant Tumours of the Breast*. Ed. P. Denoix and C. Rouquette. Basel: Karger. p. 51.

BLOOM, H. J. G. (1965) The Influence of Delay on the Natural History of Breast Cancer. *Br. J. Cancer*, **19**, 228.

BLOOM, H. J. G., RICHARDSON, W. W. & FIELD, J. R. (1970) Host Resistance and Survival in Carcinoma of the Breast. A Study of 104 Cases of Medullary Carcinomas in a Series of 1411 Cases of Breast Cancer Followed for 20 years. *Br. med. J.*, ii, 181.

BLOOM, H. J. G. (1971) Impact of Tumor Grade and Host Resistance. *Cancer, N.Y.*, **28**, 1580.

CUTLER, S. J., BLACK, M. M., FRIEDELL, G. H., VIDORE, R. A. & GOLDENBERG, I. S. (1966) Prognostic Factors in Cancer of the Female Breast. II Reproducibility of Histological Classification. *Cancer., N.Y.*, **19**, 75.

CUTLER, S. J., BLACK, M. M., MORK, T., HARVEI, S. & FREEDMAN, C. (1969) Further Observations on Prognostic Factors in Cancer of the Female Breast, *Cancer, N.Y.*, **24**, 653.

GALLAGER, H. S. & MARTIN, J. E. (1969) Early Phases in the Development of Breast Cancer. *Cancer, N.Y.*, **24**, 1170.

HAMLIN, I. M. E. (1968) Possible Host Resistance in Carcinoma of the Breast. A Histological Study. *Br. J. Cancer*, **22**, 383.

KREYBERG, L. & CHRISTIANSEN, T. (1953) The Prognostic Significance of Small Size in Breast Cancer. *Br. J. Cancer*, **7**, 37.

KUSAMA, S., SPRATT, J. S., DONEGAN, W. L., WATSON, F. R. & CUNNINGHAM, C. (1972) The Gross Rates of Growth of Human Mammary Carcinoma. *Cancer, N.Y.*, **30**, 594.

McDIVITT, R., STEWART, F. & BERG, J. W. (1968) Tumors of the Breast. *Atlas of Tumour Pathology*. Armed Forces Institute of Pathology.

MOORE, O. S. & FOOTE, F. W. (1949) The Relative Favourable Prognosis of Medullary Carcinoma of the Breast. *Cancer, N.Y.*, **2**, 635.

RICHARDSON, W. W. (1956) Medullary Carcinoma of the Breast. *Br. J. Cancer*, **10**, 415.

SLACK, N. H., BLUMENSON, L. E. & BROSS, I. D. J. (1969) Therapeutic Implication from a Mathematical Model Characterizing the Course of Breast Cancer. *Cancer, N.Y.*, **24**, 960.

SMOLAK, K., KOLODZIEJSKA, H. & URBAN, A. (1968) Investigation on the Relationship Between the Clinical Course of Mammary Carcinoma and the Morphological Features of the Stroma. *Pol. med. J.*, **7**, 162. (Translated from Nowotwory XVII, No. 2/1967.)

TOUGH, I. C. K., CARTER, D. C., FRAZER, J. & BRUCE, J. (1969) Histological Grading of Breast Cancer. *Br. J. Cancer*, **23**, 294.

WOLFF, B. (1966) Histological Grading in Carcinoma of the Breast. *Br. J. Cancer*, **20**, 36.